# Preliminary experience of stent-assisted coiling of wide-necked intracranial aneurysms with a single microcatheter

**DOI:** 10.1186/s12883-019-1470-8

**Published:** 2019-10-22

**Authors:** Keun Young Park, Chang Ki Jang, Jae Whan Lee, Dong Joon Kim, Byung Moon Kim, Joonho Chung

**Affiliations:** 1 Department of Neurosurgery, Severance Hospital, Yonsei University College of Medicine, 50-1, Yonsei-ro, Seodaemun-gu, Seoul, 03722 Republic of Korea; 2 Department of Radiology, Severance Hospital, Yonsei University College of Medicine, Seoul, Republic of Korea; 3 Severance Institute for Vascular and Metabolic Research, Yonsei University College of Medicine, Seoul, Republic of Korea

**Keywords:** Endovascular treatment, Intracranial aneurysm, Neuroform atlas, Stent assisted coiling, Stent through

## Abstract

**Background:**

The purpose of this study was to report our preliminary experience of stent-assisted coiling (SAC) of wide-necked intracranial aneurysms with a single microcatheter in patients with parent arteries that were small-caliber, with stenosis, or a very tortuous course.

**Methods:**

Between March 2018 and December 2018, we treated 394 aneurysms in 359 patients with endovascular treatment. Among 197 aneurysms treated by SAC, there were 16 cases (all wide-necked unruptured aneurysms) treated by SAC with a single microcatheter and a Neuroform Atlas stent. Follow-up angiography was performed at 6 to 12 months after SAC, and clinical follow-up was performed from 6 to 12 months in all patients.

**Results:**

The reasons for SAC with a single 0.0165-in. microcatheter were small-caliber (*n* = 4), stenosis (*n* = 2), and very tortuous course (*n* = 10) of the parent arteries. There was no complication related to delivering or deploying the Neuroform Atlas stent as well as no failure of selecting aneurysm by cell-through technique. All patients had a modified Rankin score of 0 at discharge and at follow-up. Initial angiographic results showed six cases (37.5%) of complete occlusion. In follow-up angiographies, 12 cases (75.0%) achieved compete occlusion.

**Conclusion:**

When performing SAC of wide-necked intracranial aneurysms in parent arteries with small-caliber, stenosis, or a very tortuous course, cell-through SAC using a single microcatheter and a Neuroform Atlas stent within a 5 Fr- (or smaller) guiding or intermediate catheter might be a useful option.

## Background

Despite accumulated experience and improved understanding of the devices, endovascular treatment of wide-necked intracranial aneurysms continues to be difficult to perform due to limitations arising from cerebrovascular anatomical structures [1]. When performing stent-assisted coiling (SAC), a 6-Fr or larger guiding catheter is usually needed to support two microcatheters; one for stent delivery and the other for coil delivery. A guiding catheter is placed in the internal carotid artery (ICA) for anterior circulation aneurysms and in the vertebral artery (VA) for posterior circulation aneurysms. However, when placing a 6-Fr guiding catheter it is possible to encounter ICAs or VAs that are small-caliber, exhibit stenosis, or a very tortuous course. In such cases, a smaller (5-Fr or less) guiding catheter or an intermediate catheter can be placed to support a microcatheter, but not two microcatheters. Thus, SAC for wide-necked intracranial aneurysms cannot be performed with two microcatheters if they have proximal parent arteries exhibiting a small-caliber, stenosis, or a very tortuous course.

The purpose of this study was to report our preliminary experience with cell-through SAC for wide-necked intracranial aneurysms with a single 0.0165-in. microcatheter in patients with parent arteries of small-caliber, with stenosis, or exhibiting a very tortuous course.

## Methods

### Patient selection

This retrospective study was approved by our Institutional Review Board, and the requirement for informed consent was waived. From March 2018, the Neuroform Atlas (Stryker Neurovascular, Fremont, USA) has been approved and used in our country. Since then, we treated 394 aneurysms (336 unruptured and 58 ruptured) in 359 patients with endovascular treatment. Among 197 aneurysms treated with SAC, there were 16 cases (all wide-necked unruptured aneurysms) treated with cell-through SAC using a single microcatheter, Excelsior SL-10 (Stryker Neurovascular). Among the 58 ruptured aneurysms during the same period, we tried to perform endovascular treatment other than using stents due to our results of stent-assisted coiling in ruptured aneurysms [2].

All the 16 cases underwent digital subtraction angiography (DSA) and rotational angiography (Philips Allura FD20 Clarity System, Philips Medical Systems, Best, The Netherlands) with three-dimensional reconstructions to characterize aneurysm and parent artery anatomy (Allura 3D-RA workstation, Philips Medical Systems). From these DSA results, proximal parent arteries, including the extracranial ICA or VA, were determined to use a smaller (< 6-Fr) guiding catheter due to its small-caliber, stenosis, or tortuous course. Wide-necked aneurysms were defined as having a neck width ≥ 4 mm or a dome-to-neck ratio of < 2 [2, 3].

### Endovascular treatment

The treatment decision was made by agreement of microvascular neurosurgeons and neurointerventionalists. All SAC were performed under general anesthesia. A 6-Fr femoral sheath was inserted into the right common femoral artery. A 5-Fr guiding catheter Envoy (Codman Neurovascular, Miami Lakes, FL, USA) was positioned in the ICA or VA. In some cases, a 6-Fr Shuttle (Cook Medical, Bloomington, IN, USA) was positioned in the common carotid artery or the subclavian artery followed by a 5-Fr intermediate catheter (Sofia, MicroVention, Tustin, CA, USA) in the ICA or VA. Intravenous systemic heparin (50 U/kg) was administered while placing the guiding catheter. Patients took a daily dose of 75 mg of clopidogrel and 100 mg of aspirin for more than 7 days before the SAC procedure.

**Fig. 1:**
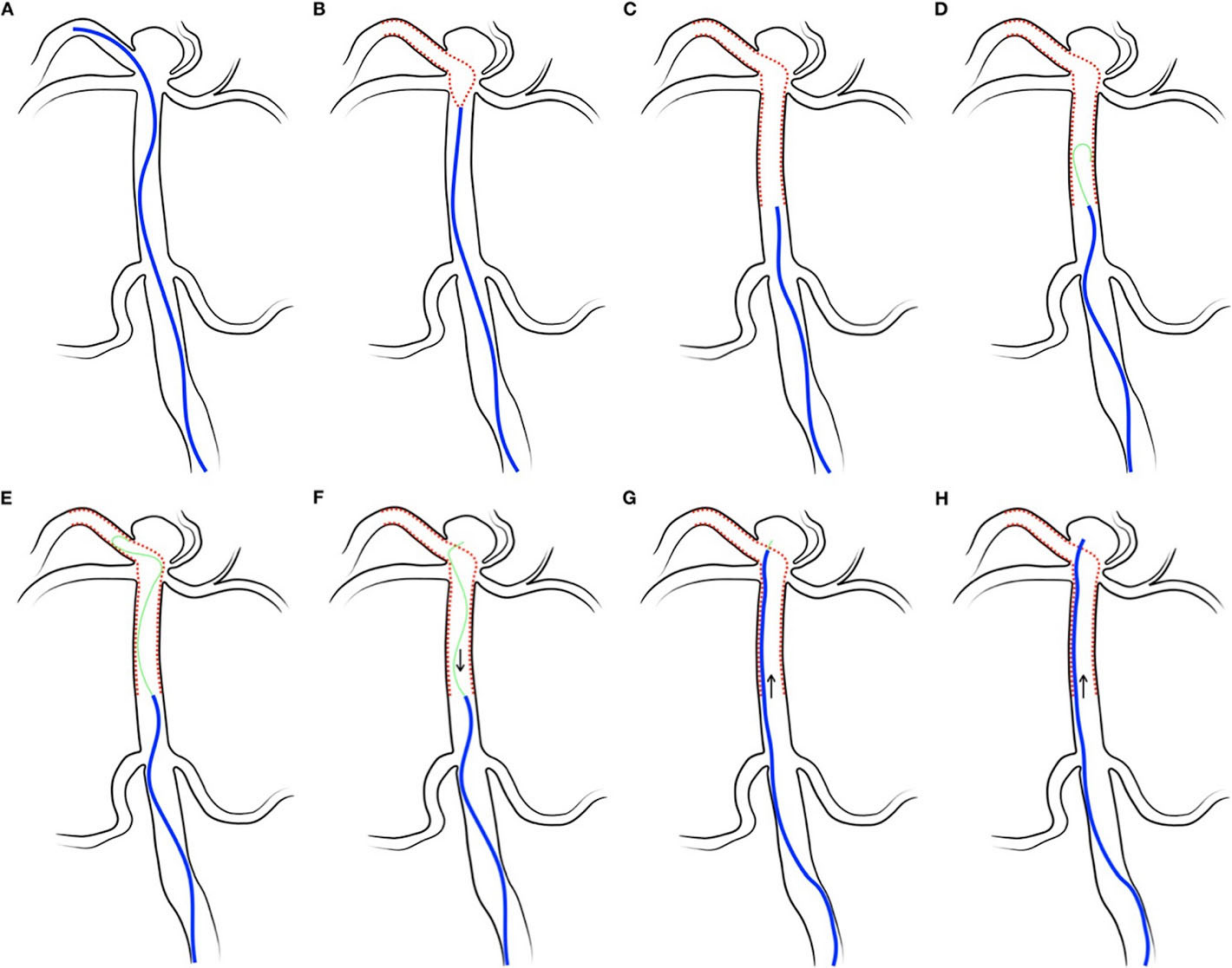
**a** A microcatheter, Excelsior SL-10, was navigated to parent vessels for delivering the Neuroform Atlas stent to cover the wide-neck of the aneurysm. **b** The Neuroform Atlas stent was being deployed. **c** The microcatheter was placed just proximal to the Neuroform Atlas deployed. **d** When the microwire could not be advanced through the stent or was stuck by the struts, a 0.014-in. microwire using its tip flexed into a loop (microwire looping technique) provided easy navigation through the stent, and (**e**) placed just distal to the aneurysm neck. **f** Make the tip of the microwire point to the aneurysm neck by using torque and pull the microwire back slowly. Then the tip can be caught by the cell of the stent and inserted into the aneurysm sac. **g** Following the microwire, the microcatheter was advanced to the neck of the aneurysm. **h** When the microcatheter could be advanced through the stent or was stuck in the struts, the microcatheter could overcome the thin-flexible struts and be advanced into the aneurysm by pushing the microcatheter very gently against the strut while torqueing and withdrawing the microwire simultaneously

An Excelsior SL-10 microcatheter was navigated to parent vessels to deliver the Neuroform Atlas stent to cover the wide-neck of the aneurysm (Fig. 1a). After deploying the Neuroform Atlas (Fig. 1b), the microcatheter was placed just proximal to the Neuroform Atlas (Fig. 1c). After removing the pusher wire of the Neuroform Atlas, a microwire was advanced through the stent proximal closed-cell segment. When the microwire could not be advanced through the stent or was stuck by the struts, a 0.014-in. microwire with its tip flexed into a loop (microwire looping technique) provided easy navigation through the stent (Fig. 1d) and was placed just distal to the aneurysm neck (Fig. 1e). Using torque, we advanced the tip of the microwire point to the aneurysm neck and pulled the microwire back slowly. Then, the tip was caught by the cell of the stent and was inserted into the aneurysm sac (Fig. 1f). Following the microwire, the microcatheter was advanced to the neck of the aneurysm (Fig. 1g). When the microcatheter could not be advanced through the stent or was stuck in the struts, pushing the microcatheter very gently against the strut while torqueing and withdrawing the microwire simultaneously allowed the microcatheter to overcome the thin-flexible struts and advance into the aneurysm (Fig. 1h). Then, coil embolization with the cell-through technique was conducted.

After the procedure, patients were prescribed 75 mg of clopidogrel daily for 3 months and 100 mg of aspirin daily for at least 12 months. Magnetic resonance imaging was routinely performed within 24 h after SAC for all patients and included diffusion-weighted imaging, T2-weighted imaging, fluid-attenuated inversion recovery, and gradient echo imaging.

### Clinical and radiographic outcomes

Patient and aneurysm characteristics, procedure-related complications, angiographic results, and clinical outcomes were reviewed retrospectively. Procedure-related events were defined as any (symptomatic or asymptomatic) events during the procedure. Immediate post-coiling and follow-up angiographic results were evaluated by 2 independent investigators who were not involved in patient care and categorized according to the modified Raymond-Roy classification (Class I: complete obliteration, Class II: residual neck, Class IIIa: residual aneurysm: contrast within coil interstices, Class IIIb: residual aneurysm: contrast along aneurysm wall) [4]. Clinical outcomes were assessed using the modified Rankin Scale (mRS) and evaluated at the time of discharge and out-patient follow-up by an independent investigator who was not involved in patient care.

### Statistical analysis

All statistical analyses were performed using IBM SPSS Statistics version 22.0 (IBM, Armonk, New York, USA) in consultation with a biostatistician. Mann-Whitney U test was used for numeric variables. Chi-square test or Fisher exact test were used for nominal variables. A *P* value less than 0.05 for a 95% confidence interval was considered statistically significant.

## Results

The data and outcomes from all 16 cases are summarized in Table 1. There were 4 men and 12 women. The ages of the patients ranged from 50 to 74 years, with a mean of 62.8 years. Six aneurysms were located in the anterior communicating artery, five were in the middle cerebral artery, and five were at the basilar bifurcation. All 16 aneurysms were classified as small (< 10 mm) with a mean maximal length of 4.9 mm and a mean neck size of 4.0 mm. The reasons for using the cell-through technique were parent artery small-caliber (*n* = 4), stenosis (*n* = 2), or tortuous course (*n* = 10). There were no complications related to delivering or deploying the Neuroform Atlas stent as well as no failures in selecting the aneurysm by cell-through technique. However, two asymptomatic procedure-related events, left proximal VA dissections, occurred during the placement of a guiding catheter (Case 5 & 10).

**Table 1:** All the data and outcomes of patients

Pt.	Sex	Age	Aneurysm location	Length (mm)	Neck size (mm)	Reason for cell-through technique	Stent size (mm)	Procedure-related event	Initial angiographic results	mRS at discharge	Follow-up period (months)	Follow-up angiographic results	mRS at follow-up
1	F	62	MCA bif., Left	4.3	3.4	Tortuosity	3.0 × 15	No	I	0	12	I	0
2	F	70	Acom, left	4.4	3.3	Stenosis	3.0 × 15	No	II	0	12	I	0
3	F	69	M1, left	3.9	3.8	Tortuosity	4.0 × 21	No	IIIa	0	11	II	0
4	F	67	Acom, right	5.1	3.9	Tortuosity	3.0 × 15	No	I	0	11	I	0
5	F	55	BA bif.	4.9	4.1	Small-caliber	4.0 × 21	Yes	II	0	10	I	0
6	M	50	Acom, left	4.9	3.7	Small-caliber	3.0 × 15	No	IIIa	0	10	IIIa	0
7	F	60	M1, left	4.6	3.9	Stenosis	3.0 × 15	No	II	0	10	I	0
8	M	64	Acom, right	4.8	3.9	Small-caliber	3.0 × 15	No	I	0	9	I	0
9	F	58	BA bif.	5.2	4.1	Tortuosity	4.0 × 21	No	II	0	8	I	0
10	F	61	BA bif.	4.4	3.5	Tortuosity	4.0 × 21	Yes	I	0	8	I	0
11	F	58	BA bif.	5.8	4.0	Tortuosity	4.0 × 21	No	II	0	8	I	0
12	F	69	BA bif,	8.3	5.5	Small-caliber	4.0 × 21	No	I	0	6	I	0
13	F	74	Acom, left	4.7	3.0	Tortuosity	3.0 × 15	No	I	0	6	I	0
14	M	64	A1, left	4.2	4.7	Tortuosity	3.0 × 15	No	II	0	6	II	0
15	F	58	M1, left	4.4	4.1	Tortuosity	3.0 × 15	No	II	0	6	I	0
16	M	65	M1, right	4.6	4.8	Tortuosity	3.0 × 15	No	IIIa	0	6	II	0

*Acom* Anterior communicating artery, *BA* Basilar artery, *bif*. Bifurcation, *CO* Complete occlusion, *F* Female, *M* Male, *MCA* Middle cerebral artery, *mRS* Modified Rankin Scale, *NA* Not applicable, *NR* Neck remnant, *PO* Partial occlusion

Angiographic results were classified according to the Raymond-Roy classification

Magnetic resonance angiography (MRA) or DSA were performed in all patients at 6 to 12 months after SAC, and clinical follow-up was performed from 6 to 12 months (mean 8.7 months) after SAC. All patients had mRS 0 at discharge and at follow-up. Initial angiographic results according to the Raymond-Roy classification showed six cases (37.5%) of class I, seven cases (43.8%) of class II, and three cases (18.8%) of class IIIa. In follow-up angiographies, 12 cases (75.0%) showed class I, three cases (18.8%) showed class II, and one case (6.3%) showed class IIIa.

Comparing to 181 cases undergone SAC using standard two microcatheters during the same period, there were no significant differences in baseline characteristics, procedure-related complications (asymptomatic and symptomatic), and angiographic results (Table 2).

**Table 2:** Comparison of baseline characteristics, procedure-related complications, and angiographic outcomes between 16 cases undergone Neuroform Atlas stent-assisted coiling using a single microcatheter and 181 cases undergone stent-assisted coiling using two or more microcatheters

Variables	Atlas using a single microcatheter (*n* = 16)	SAC using two or more microcatheters (*n* = 181)	*P* value
Age (mean ± SD, years)	62.8 ± 6.2	59.4 ± 10.2	0.525^a^
Female (n, %)	12 (75.0)	136 (75.1)	0.913
Hypertension (n, %)	6 (37.5)	69 (38.1)	0.846
Diabetes (n, %)	2 (12.5)	21 (11.6)	0.782
Smoking (n, %)	2 (12.5)	26 (14.4)	0.636
Aneurysm location (n, %)			0.253
Anterior circulation	11 (68.8)	145 (80.1)	
Posterior circulation	5 (31.3)	36 (19.9)	
Neck size (mean ± SD, mm)	4.0 ± 0.6	4.5 ± 2.5	0.225^a^
Aneurysm size (mean ± SD, mm)	4.9 ± 1.0	5.9 ± 2.8	0.175^a^
Stent used (n, %)			0.061
Neuroform Atlas	16 (100)	120 (66.3)	
Enterprise2	0 (0)	26 (14.4)	
LIVS or LVIS Jr.	0 (0)	32 (17.7)	
Procedure-related complications (n, %)			0.112
Asymptomatic	2 (12.5)	9 (5.0)	
Symptomatic	0	1 (0.6)	
Initial angiographic results (n, %)			0.088
Raymond-Roy I	6 (37.5)	82 (45.3)	
Raymond-Roy II	7 (43.8)	64 (35.4)	
Raymond-Roy III	3 (18.8)	35 (19.3)	
Follow-up angiographic results (n, %)	n = 16	*n* = 155	0.814
Raymond-Roy I	12 (75.0)	115 (74.2)	
Raymond-Roy II	3 (18.8)	31 (20.0)	
Raymond-Roy III	1 (6.3)	9 (5.8)	

^a^Mann-Whitney U test. *SAC* Stent-assisted coiling, *SD* Standard deviation

### Case illustration

#### Case 5

**Fig. 2:**
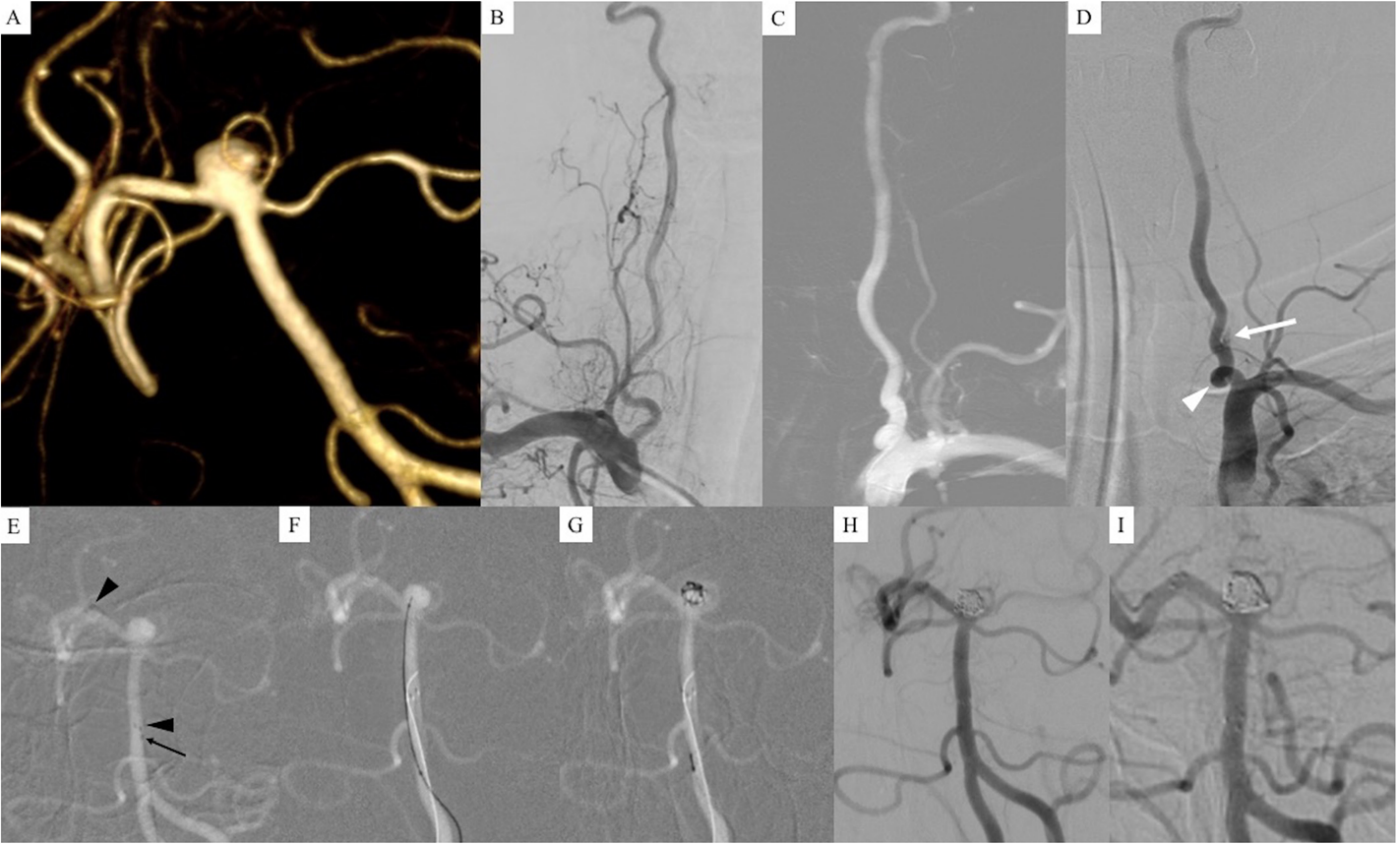
**a** 3D-DSA demonstrated a wide-necked (4.1 mm) basilar bifurcation aneurysm sized 4.9 mm with a daughter sac on the left lateral side of the sac. **b** and **c** Left proximal vertebral artery (VA) was dominant compared to the right VA so that we decided to select the left VA for a guiding catheter during endovascular treatment. **d** A 6-Fr guiding catheter could not pass through the tortuosity of the left VA at the proximal segment (a white arrowhead). There was asymptomatic dissection of the left VA (a white arrow). **e** With a 5Fr-guinding catheter in the right VA, a single microcatheter (Excelsior SL-10 straight) was navigated to the right posterior cerebral artery and we deployed a Neuroform Atlas stent (3.0 mm × 21 mm) from the right posterior cerebral artery to the basilar artery (black arrowheads). After deploying the stent, the microcatheter was placed just proximal to the stent deployed (a black arrow). **f** A 0.014-microwire and the same microcatheter were navigated easily through the stent to the aneurysm. **g** Coiling was performed. **h** The initial angiographic result showed a neck remnant of the aneurysm. (**i**) On 6-month follow-up angiography, the aneurysm was completely occluded

A 55-year-old woman presented with a long-standing history of headache. Her computed tomography (CT) and CT angiography revealed no intracranial hemorrhage and a basilar bifurcation aneurysm. DSA demonstrated a wide-necked (4.1 mm) basilar bifurcation aneurysm sized 4.9 mm with a daughter sac on left lateral side of the sac (Fig. 2a). On DSA, left proximal VA was dominant compared to right VA (Fig. 2b and c) so that we decided to select left VA for a guiding catheter during endovascular treatment. However, a 6-Fr guiding catheter could not pass through a left VA tortuosity at the proximal segment (a white arrowhead in Fig. 2d). Unfortunately, there was asymptomatic dissection of the left VA (a white arrow in Fig. 2d). We changed our plan for using a 5Fr-guiding catheter in the left VA and selected the right VA, which has a smaller diameter compared to the left VA. A single microcatheter (Excelsior SL-10 straight) was navigated to the right posterior cerebral artery and we deployed a Neuroform Atlas stent (4.0 mm × 21 mm) from the right posterior cerebral artery to the basilar artery (black arrowheads in Fig. 2e). After deploying the stent, the microcatheter was placed just proximal to the deployed stent (a black arrow in Fig. 2e). After removing the stent pusher wire, a 0.014-microwire and the same microcatheter were navigated easily through the stent to the aneurysm (Fig. 2f). Coiling was performed (Fig. 2g) and initial angiographic results showed a neck remnant of the aneurysm (Fig. 2h). On the 6-month follow-up angiography, the aneurysm was completely occluded (Fig. 2i).

#### Case 7

**Fig. 3:**
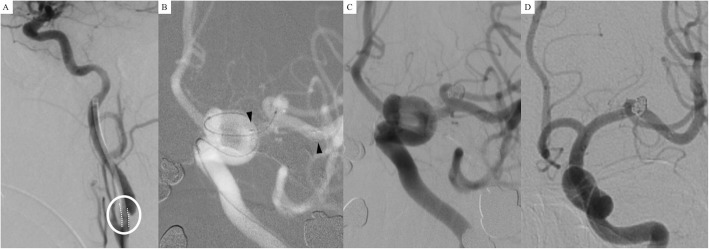
**a** A 5Fr-guiding catheter was placed in the left internal carotid artery (ICA) with stenosis of about 40% in the left carotid bulb. The guiding catheter seemed to be stuck in the stenotic segment of the ICA (white dots inside of a white circle). **b** A single microcatheter (pre-shaped Excelsior SL-10 45 degree) was steam-shaped into a “Z” shape and navigated to the left MCA. We deployed a Neuroform Atlas stent (3.0 mm × 15 mm) in the left M1 (black arrowheads) and selected the aneurysm with the same microcatheter using the cell-through technique. **c** A neck remnant was seen in the final angiography after coiling. (**d**) On 6-month follow-up angiography, the aneurysm was completely occluded

A 60-year-old woman experienced headaches, and a left middle cerebral artery aneurysm was diagnosed on MRA. She was willing to treat the lesion aggressively because of a family history of ruptured intracranial aneurysms. DSA revealed a middle cerebral artery aneurysm approximately 4.6 mm in maximal length with a neck size of 3.9 mm and carotid stenosis of about 40% in the left carotid bulb. For endovascular treatment of the aneurysm, a 5Fr-guiding catheter was placed in the left ICA, but it seemed to be stuck in the stenotic segment of the ICA (white dots inside of a white circle in Fig. 3a). A single microcatheter (pre-shaped Excelsior SL-10 45 degree) was steam-shaped into a “Z” shape and navigated to the left MCA. We deployed a Neuroform Atlas stent (3.0 mm × 15 mm) in left M1 (black arrowheads) and selected the aneurysm with the same microcatheter using the cell-through technique (Fig. 3b). A neck remnant resulted in the final angiography after coiling (Fig. 3c). On 6-month follow-up angiography, the aneurysm was completely occluded (Fig. 3d).

#### Case 9

**Fig. 4:**
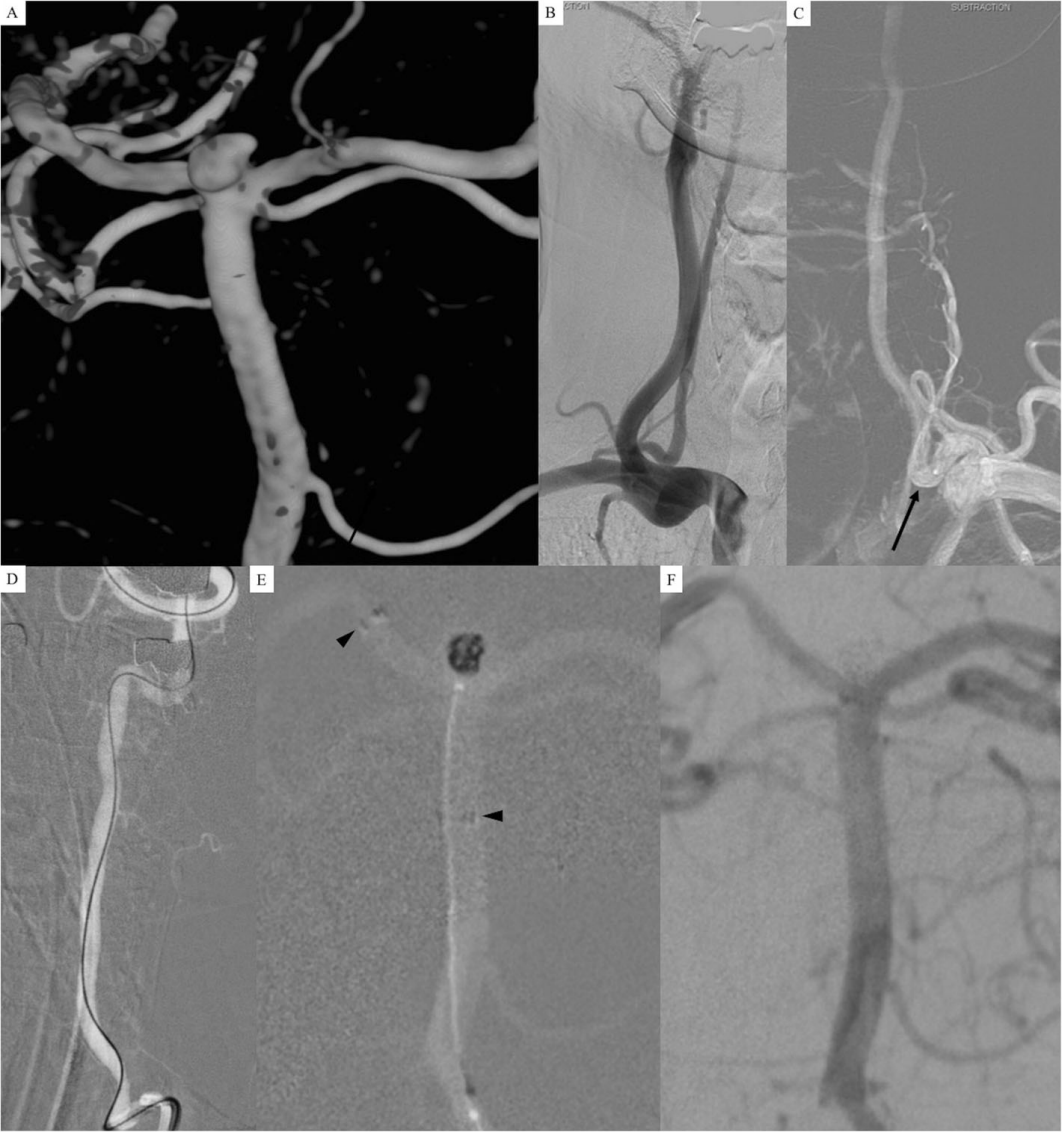
**a** 3D-DSA showed a basilar artery bifurcation aneurysm sized 5.2 mm with a spiculate on the left posterior-lateral direction. **b** and **c** Left proximal vertebral artery (VA) was dominant compared to the right VA so that we decided to select the left VA for a guiding catheter. Neither a 6-Fr nor 5-Fr guiding catheter went up through the tortuous segment of the left proximal VA (a black arrow). **d** We placed a 5-Fr guiding catheter in the left proximal VA and a single microcatheter (pre-shaped Excelsior SL-10 45 degree) was navigated from the VA to the right posterior cerebral artery. **e** We deployed a Neuroform Atlas stent (3.0 mm × 21 mm) from the right posterior cerebral artery to the basilar artery (black arrowheads). We selected the aneurysm with the same microcatheter through the stent struts and coiling was performed. **f** Initial angiographic results showed a neck remnant of the aneurysm

A 58-year-old woman with sudden bursting severe headache was admitted. She was alert and there was no intracranial hemorrhage on her brain CT and MRI. DSA showed a wide-necked (4.1 mm) basilar artery bifurcation aneurysm sized 5.2 mm with a speculate in the left posterior-lateral direction (Fig. 4a). We planned to treat the aneurysm by using SAC. On DSA, the left proximal VA was dominant compared to the right VA (Fig. 4b and c) so we decided to select the left VA for a guiding catheter during endovascular treatment. Neither a 6-Fr nor 5-Fr guiding catheter went up through the left proximal VA (a black arrow) tortuous segment. We placed a 5-Fr guiding catheter in the left proximal VA and a single microcatheter (pre-shaped Excelsior SL-10 45 degree) was navigated from the VA to the right posterior cerebral artery (Fig. 4d). We deployed a Neuroform Atlas stent (4.0 mm × 21 mm) from the right posterior cerebral artery to the basilar artery (black arrowheads). We selected the aneurysm with the same microcatheter through the stent struts and coiling was performed (Fig. 4e). Initial angiographic results showed a neck remnant of the aneurysm (Fig. 4f).

## Discussion

In the present study, we described 16 cases of SAC treated with the cell-through technique using a single microcatheter (Excelsior SL-10) and a Neuroform Atlas stent in patients with unruptured wide-necked intracranial aneurysms. There was no complication related to delivering or deploying the Neuroform Atlas stent as well as no failure of selecting aneurysms by the cell-through technique. Not the cell-through technique but the jailing technique is our routine method for SAC. However, we found that this cell-through technique might be feasible in patients with small-caliber, stenotic, or very tortuous parent arteries.

Recently, complicated wide-necked aneurysms rather than simple saccular aneurysms have been treated by endovascular methods, but surgeons need more than a single microcatheter to treat those aneurysms. Multiple microcatheter, balloon-assisted, or stent-assisted techniques are needed. For that purpose, surgeons also need a strong and firm guiding catheter to support those devices. A guiding catheter has a very important role in supporting devices being delivered to tortuous intracranial vessels when performing endovascular treatments, including microcatheters, coils, balloons, or stents [5–7]. However, when faced with an ICA or VA of small-caliber, with stenosis, or a very tortuous course, it can be difficult to place a 6Fr- or larger guiding catheter to perform multiple microcatheter, balloon-assisted, or stent-assisted techniques. In such cases, a 5Fr- (or smaller) guiding catheter or intermediate catheter would be placed and there will be only one option to use a single microcatheter. Thus, in those situations, we tried to perform SAC with a cell-through technique for wide-necked intracranial aneurysms by using a single microcatheter and a Neuroform Atlas stent.

The Neuroform Atlas stent is the latest version of the Neuroform series reinforcing its navigability in a smaller microcatheter, stability to the vessel wall (high radial force), accurate placement (ease of use with very low foreshortening), wall apposition, conformability, and coil protection. The stent can be delivered via a 0.0165-in. microcatheter, the smallest profile, allowing surgeons to perform SAC with a single microcatheter, a sequential technique where the same microcatheter that is used to place the stent is then used to coil the aneurysm. The cell size of the Neuroform Atlas stent has been reduced compared to that of its predecessor, the Neuroform stent, to achieve better coil protection in aneurysms and to allow the use of smaller coils. Additionally, the width of struts has been reduced so that the flexibility and conformability of the stent are improved. Furthermore, the segmental opening of the struts allows stable positioning and direct vessel wall apposition is obtained by anchoring the stent after the first rows of struts exit the microcatheter. There have been only a few reports on SAC with Neuroform Atlas stents. Most of them reported their preliminary experience with using Neuroform Atlas stents [6–13]. From their experiences with 27 consecutive patients one study concluded that Neuroform Atlas SAC is a feasible way to treat ruptured and unruptured wide-necked aneurysms that are not amenable to conventional coiling or clipping [9]. Aneurysm occlusion and favorable clinical outcomes were consistent with previously reported rates for SAC of wide-neck aneurysms using other devices [9]. From their experiences with 37 aneurysms in 36 patients, another report concluded that deployment of the Neuroform Atlas stent was a safe and effective method for the treatment of wide-necked aneurysms [8].

Although it is not always easy to advance a microcatheter into an aneurysm using SAC with the cell-through technique, there are some advantages in the use of the Neuroform Atlas stent for the cell-through technique. (1) The closed cells at the proximal end with flaring end give good navigability and enhance the cell-through technique by passing a microwire in the stent lumen easily. When a microwire cannot be advanced through the stent or is stuck in the struts, a microwire looping technique (Fig. 1d and e) provides easy navigation through the inside of the stent lumen. (2) Its open-cell design with both ends flared gives good stability to the vessel wall and wall apposition. It is easy for microcatheters and microwires to pass through the inside of the stent without irritating the struts. Otherwise, microwires cannot be navigated through the inside of the stent. Microwires may go out of the stent lumen, between the struts and the vessel wall, then come back into the stent lumen again. (3) The struts are flexible and reduce stent width. When microcatheter tips cannot be advanced into the aneurysm due to being stuck in the struts, it is possible to overcome the situation by navigating a 0.014-in. microwire through the cell followed by advancement of the microcatheter over the microwire. Pushing the microcatheter very gently against the strut, withdrawing the microwire tip in the microcatheter allows the microcatheter to advance into the aneurysm while overcoming the thin and flexible struts (Fig. 1g and h). (4) It can be delivered via a 0.0165-in. microcatheter that can be easily placed intra-stent space through the pusher wire and easily navigated into an aneurysm sac due to a smaller size of caliber and a less ledge effect. Previous stents can be delivered via a 0.027- or 0.021-in. microcatheter that was difficult to be placed into an aneurysm sac due to a relatively larger size of its caliber and a ledge effect. In some situations, exchange by 0.017 or 0.0165 microcatheter was mandatory in order to use a 0.027- or 0.021-in. microcatheter. A benefit of this cell-through SAC with a single microcatheter method is the possibility to perform SAC using 5Fr-guiding or intermediated catheters, and even when using a 4Fr-intermediate catheter. Another benefit was easy navigation through very tortuous vessels. And, once the aneurysm selection was done by the cell-through technique, then the microcatheter was stuck within a cell of the stent, becoming very stable while inserting coils. Thus, patients with small-caliber, stenotic, or very tortuous parent arteries (ICA or VA) can be good candidates for this technique.

There are some other techniques to perform SAC rather than this cell-through technique. We could perform SAC using two parent arteries with two 5Fr-guiding catheters in each parent artery. First, coil embolization with horizontal stenting could be performed crossing the Circle of Willis [14–19]. An aneurysm on the anterior communicating artery or the ICA bifurcation could be treated using a 5Fr-guding catheter each in bilateral ICAs. An aneurysm in the basilar artery aneurysm could be treated using a 5Fr-guding catheter in each ICA and VA. Second, we could perform SAC using a 5Fr-guiding catheter in bilateral VAs to treat aneurysms in the posterior circulation [20, 21]. An aneurysm on the posterior inferior cerebellar artery could be treated using bilateral VAs. However, none of these are universal techniques for SAC. Those techniques can be applied on a case by case basis. Thus, we believed that this cell-through SAC technique with a single microcatheter and a Neuroform Atlas stent might provide neurointerventionists one more option to treat wide-necked intracranial aneurysms with small-caliber, stenotic, or very tortuous parent arteries.

Other low-profile neurovascular stents have been introduced and used clinically, including the braided LVIS Jr. or LEO Baby (Balt, Montmorency, France) that are compatible with a 0.0165-in. microcatheter [22]. These devices can be placed through the same microcatheter needed for subsequent coiling. Thus, they may be used in this cell-through SAC. However, LEO Baby is not available in our country. We have experienced cell-through SAC using LVIS Jr., not intentionally with a single microcatheter but secondarily due to accidental kick-back of the microcatheter while performing SAC with a jailing technique. Thus, we have not performed cell-through SAC using LVIS Jr. with the same single microcatheter.

This study was a retrospective study with a small case series so that the effects of possible selection bias cannot be excluded. However, the cases in the present study could not be done by usual SAC using two microcatheters. Another technique, such as cell-through SAC using a single microcatheter, was inevitable. Thus, it was difficult to have a true control group for evaluating the safety and efficacy of this cell-through SAC technique. In addition, a longer follow-up period is needed to evaluate the true safety and efficacy of this technique. Furthermore, it was challenging to describe the geometry and tortuosity of cerebral arteries in 3D space, because determining uniform measurement standards was difficult. In the present study, we grossly categorized the vessel conditions; parent arteries of small-caliber, with stenosis, or a very tortuous course.

## Conclusions

When performing SAC in patients with wide-necked intracranial aneurysms, rather than struggling to place a 6Fr-guiding catheter in small-caliber, stenotic, or very tortuous parent arteries (ICA or VA), the cell-through SAC might be a useful option by using a single microcatheter and a Neuroform Atlas stent within a 5Fr- (or smaller) guiding or intermediate catheter.

## Data Availability

The datasets used and analyzed during the current study are available from the corresponding author on reasonable request.

## Abbreviations

CT: Computed tomography
DSA: Digital subtraction angiography
ICA: Internal carotid artery
MRA: Magnetic resonance angiography
mRS: Modified Rankin Scale
SAC: Stent-assisted coiling
VA: Vertebral artery
